# Developing an Undergraduate Public Health Introductory Core Course Series

**DOI:** 10.3389/fpubh.2018.00155

**Published:** 2018-05-28

**Authors:** Denise C. Nelson-Hurwitz, Michelle Tagorda, Lisa Kehl, Opal V. Buchthal, Kathryn L. Braun

**Affiliations:** ^1^Office of Public Health Studies, University of Hawai‘i at Mānoa, Honolulu, HI, United States; ^2^Health Careers Opportunity Program, Student Equity, Excellence and Diversity, University of Hawai‘i at Mānoa, Honolulu, HI, United States

**Keywords:** public health education, bachelors of public health, undergraduate public health, undergraduate education, curriculum development, pedagogy

## Abstract

The number of undergraduate public health education programs is increasing, but few publications provide examples of introductory public health courses that provide foundational knowledge and meet 2016 Council on Education in Public Health (CEPH) accreditation standards. This article presents the development and testing of a three-course, introductory series in public health at the University of Hawai‘i at Mānoa (UHM). Development was informed by best pedagogical practices in education, web review of existing programs, literature review, key informant interviews, and accreditation standards. Student mastery of required concepts, domains, and competencies is assessed through testing and class assignments. Data from course evaluations, students' exit questionnaires at graduation, and faculty feedback were used to continuously evolve and adapt the curriculum. The three-course series—including Introduction to Public Health, Public Health Issues in Hawai‘i, and Introduction to Global Health—was designed to provide incoming undergraduate public health students with a foundation in local, national, and global public health concepts and domains, while improving their skills in public health communication and information literacy. Data from class assignments, examinations, and later coursework suggest students are mastering the course materials and gaining required competencies. Data from course evaluation and exit questionnaires suggest that the students appreciate the series' approach and the challenge to apply course concepts locally and globally in subsequent courses. This foundational public health series provides a model for an introductory course series that can be implemented with existing resources by most programs, meets the new CEPH requirements, is well-received by students, and prepares students well for upper-division public health courses.

## Background and rationale

Previous investigators and administrators have written about the importance of public health education at the undergraduate level (1–4). Expanding baccalaureate training in public health is important because it will increase the number of individuals ready to join the public health workforce, nationally and worldwide, and it will increase the public health knowledge and engagement of all college graduates, regardless of their ultimate career choice (5–7).

In the United States, conferrals of undergraduate degrees in public health have increased rapidly from roughly 758 conferrals in 1992, to 9,525 conferrals in 2014^1^ The development, and offering, of educational programs in public health are guided by standards established by the Council on Education in Public Health (CEPH) (8). These standards outline nine foundational domains, 12 cross-cutting concepts, and two competencies to be mastered by students. The nine foundational domains include (1) history and philosophy of public health; (2) methods of data collection, use, and analysis; (3) concepts of population health; (4) underlying science of human health; (5) social, behavioral, biological, environmental determinants of health; (6) concepts of project implementation; (7) characteristics of health care systems; (8) concepts of legal, ethical, economic, and regulatory dimensions of health care; and (9) concepts of public health communication. The 12 cross-cutting concepts include: (1) advocacy for protection and promotion of the public's health; (2) community dynamics; (3) critical thinking and creativity; (4) cultural contexts; (5) ethical decision making; (6) a personal work ethic; (7) networking; (8) organizational dynamics; (9) professionalism; (10) research methods; (11) systems thinking; and (12) teamwork and leadership. The two competencies require students to be able to: (1) communicate public health information, in both oral and written forms and through a variety of media, to diverse audiences; and (2) locate, use, evaluate, and synthesize information.

However, few curricular and pedagogical models exist to show how introductory public health courses can ensure student exposure to, and mastery of, the CEPH-required elements. Yeatts (9) reported on an introductory course in public health that incorporated critical component elements (CCEs) identified by the Association of Schools and Programs of Public Health (ASPPH)^2^, along with Liberal Education and America's Promise (LEAP) learning objectives (10, 11). These related to inquiry and analysis, critical and creative thinking, written and oral communication, quantitative literacy, information literacy, teamwork, problem solving, social responsivity, and integrative learning. The CECs and LEAP learning objectives preceded, and were incorporated into, the 2016 CEPH standards. Two additional publications describe public health capstone courses outlining how they reflect the recommended CCE, LEAP, and (ultimately) CEPH learning objectives and competencies (12, 13).

The University of Hawai‘i at Mānoa (UHM) is a minority-serving, land-, sea-, and space-grant institution with a mission to provide a Hawaiian place of learning and serve the people of the state. It has a student population of 17,612 individuals, of which, nearly three-quarters are undergraduates, and only about 22.2% are Caucasian^3^ Prior to the addition of the Bachelor of Arts in Public Health (BA PH) degree in 2014, public health education at UHM was offered only at the graduate level. Faculty members designing the BA PH created three courses that would introduce students to the breadth of public health. After initial development of individual courses, efforts were made to capitalize on synergistic impacts by transitioning the individual courses to function as a 3-course series. The purpose of this article is to outline how a required three-course public health introductory series was developed for undergraduates at UHM and how bachelors-specific CEPH requirements were incorporated.

## Pedagogical framework and principles

First, the program development faculty searched the literature for information on baccalaureate public health education and best practices in pedagogical techniques. They also reviewed the academic standards of existing programs. Faculty searched the web for other CEPH-accredited baccalaureate public health programs at peer institutions, with particular attention paid to institutions of long-standing, which had developed a baccalaureate degree option in a program that had traditionally offered public health degrees at the graduate level. Web searches centered on investigation of both comprehensive curriculum and individual course design.

Interviews were conducted with undergraduate faculty and staff involved in teaching comparable introductory courses in different disciplines on campus. Questions focused on instructional experiences, best practices, and suggested needs for student support infrastructure. Additional interviews were conducted with local public health community leaders, and university administration to ensure the developed courses articulated well with community needs as well as existing curricular requirements and institutional learning objectives. Evaluation processes were established for the baccalaureate degree at the individual course and program levels. Articulation of introductory core skills and concepts were linked to key CEPH criteria for foundational domains, cross-cutting concepts and experiences, and competencies (see Tables 1–**3**).

**Table 1:** Public health Bachelor's degree foundational domains (CEPH criterion D10).

	**PH 201 Introduction to PH**	**PH 202 PH Issues in Hawai‘i**	**PH 203 Introduction to Global Health**
**History and philosophy of PH as well as its core values, concepts, and functions across the globe and in society**			
Public health history	I	C	
Public health philosophy	I	C	C
Core PH values	I	C	C
Core PH concepts	I	C	C
Global functions of PH	I		C
Societal functions of PH	I	C	C
**Basic concepts, methods, and tools of PH data collection, use and analysis and why evidence-based approaches are an essential part of PH practice**			
Basic concepts of data collection	I	C	C
Basic methods of data collection	I	C	C
Basic tools of data collection	I	C	C
Data usage	I	C	C
Data analysis	I	C	C
Evidence-based approaches^a^	I		
**Concepts of population health, and the basic processes, approaches and interventions that identify and address the major health-related needs and concerns of populations**			
Population health concepts	I	C	C
Processes/approaches to identify needs and concerns of populations	I	C	C
Approaches/interventions to address needs and concerns of populations	I	C	C
**Underlying science of human health and disease, including opportunities for promoting, and protecting health across the life course**			
Science of human health and disease^b^	I		
Health promotion	I	C	C
Health protection	I	C	C
**Socioeconomic, behavioral, biological, environmental, and other factors that impact human health and contribute to health disparities**			
Socio-economic impacts	I	C	C
Behavioral impacts	I	C	C
Biological impacts	I	C	C
Environmental impacts	I	C	C
**Fundamental concepts and features of project implementation, including planning, assessment, and evaluation**			
Planning concepts and features	I	C	C
Assessment concepts and features^a^			
Evaluation concepts and features^a^			
Fundamental characteristics and organizational structures of the US health system as well as the differences between systems in other countries	I	C	C
US health system			
Comparative health systems	I		C
**Basic concepts of legal, ethical, economic, and regulatory dimensions of health care and PH policy and the roles, influences and responsibilities of the different agencies and branches of government**			
Legal dimensions	I	C	C
Ethical dimensions	I	C	C
Economical dimensions	I	C	C
Regulatory dimensions	I	C	C
Governmental roles	I	C	C
**Basic concepts of PH-specific communication, including technical and professional writing and the use of mass media and electronic technology**			
Technical writing	I	C	C
Professional writing	I		C
Mass media	I	C	C
Electronic technology	I	C	C

PH, Public Health; I, Introduced; C, Covered; Blank cells indicate that the domain is covered in an upper-level course required for the major.

a Covered in PH 480 and PH 489, which are courses in the capstone series.

b *Covered in PH 341, PH Biology*.

Initial research identified multiple recommendations and requirements for variable content and skills to be included an introductory public health course. Specifically, the program needed to incorporate CCE, LEAP, and CEPH recommendations, reflect the values of the campus, and provide local and global examples of public health (14). Program faculty also wanted to address diversity in students' learning styles and enhance learning though repetition, scaffolding, and application of concepts and skills. Individual courses were developed to meet general education and strategic campus initiatives of UHM, as well as to promote active learning among undergraduate students. To meet multiple agendas, it was determined most effective to offer an introductory series, rather than a single introductory course. It was also decided to incorporate a variety of pedagogical approaches into the introductory courses, including experiential learning techniques, and regular opportunities for student reflection, in addition to more traditional lectures, discussions, and worksheets.

Testing and class assignments were used to assess student mastery of required concepts, domains, and competencies. Data from mid-semester and final course evaluations, as well as reports from faculty members teaching upper-division BA PH courses, are used to continuously monitor and improve courses, while data from exit questionnaires and focus groups collected in the weeks prior to graduation also continue to be used to improve the curriculum.

In direct response to the two CEPH foundational competencies for baccalaureate public health information literacy (see Table **3**), lectures provide an overview of how to find, judge, and synthesize public health information. These skills are further applied in worksheets, activities, papers, and presentations. Written and oral communication skills are also emphasized. Class assignments facilitate practice of these skills, and students needing additional support are identified and referred to teaching assistants and other resources early in their academic careers, as recommended by Levy and Petrulis (15). Written communication skills are continuously assessed through assigned papers, as well as through weekly blogs required in all three courses, and these are graded using rubrics for evaluation of content and style. Specific rubrics available from authors. Weekly blog reflections include critical synthesis and application of the week's topics, discussions, and activities, and may include relevant personal opinions about the topics. Students may gain additional class participation points by responding to blog entries written by other students, thus promoting a class dialogue centered on the week's subject matter. Blogs have been demonstrated to be effective in enhancing student learning and participation, as well as helping to identify learning needs of students (16–18). Students also present orally in all three classes. All presentations are graded using a rubric that assesses both content and professional presentation skills.

Team-based experiential learning is incorporated into all three classes. This learning approach has been shown to be effective in higher education in general (19, 20), and in the teaching of public health (9), biology (21), pharmacy (22), nursing (23), and undergraduate science, technology, engineering, and mathematics courses (24).

## Learning objectives of the series

1. To provide new, incoming undergraduate public health students with a strong foundation in core public health knowledge and skills.
2. To facilitate application of these principles to local and global health settings.
3. To initiate the development of written and oral communication skills.
4. To facilitate information literacy.

## Learning environment

The series includes three courses: (1) Introduction to Public Health (PH 201), (2) Public Health Issues in Hawai‘i (PH 202), and (3) Introduction to Global Health (PH 203). The courses are intended to be taken in sequence; however, the latter two may be taken interchangeably or concurrently, allowing for a possible series spread over two semesters.

As an overview, PH 201 (Introduction to Public Health) introduces students to key concepts in public health and energizes students on their paths through public health education. The two subsequent courses, PH 202 (Public Health Issues in Hawai‘i) and PH 203 (Introduction to Global Health), facilitate application of concepts both locally and globally. For example, basic concepts of epidemiology and measures of disease frequency (i.e., incidence and prevalence) are introduced in PH 201. In PH 202, students observe how epidemiology is applied to track transmission patterns of influenza virus and the influence of tourism on local disease patterns. In PH 203, students discuss the global burden of HIV/AIDS and learn how epidemiological data are collected and applied for surveillance. The topics of the PH 201 course are intended as a broad overview of public health, including foundational concepts and skills, (for example, briefly defining and describing epidemiology). The focus of PH 202 to emphasize local public health issues was intended to engage students in the local public health community, where many of them have lived for most of their lives and intend to find employment after graduation, and to align with University system objectives as a Hawaiian place of learning. The focus on global health in PH 203 is to increase awareness of students to global challenges in public health, and the juxtaposition of those challenges and opportunities with that of the local public health environment.

Logistically, each course of the introductory core is taught in two class sessions of 75 min each, per week, by independent faculty instructors. Instructors meet periodically throughout the semester, share syllabi, and conduct a debrief meeting at the end of each semester to ensure fidelity to the introductory series and to discuss any potential gaps or unintended duplication in assignments or topics. Scheduling of the courses, especially PH 201, takes into account common times of undergraduate student availability on campus to provide more opportunities for students to enroll in the course. All three courses are offered during the fall and spring semesters. PH 201 is regularly offered in the summer, with PH 202 and PH 203 being offered in the summer session occasionally, dependent on instructor availability. Undergraduate students who want to major in public health must pass PH 201 with a B- or better. Students outside of the major are able to pass the course with a D- or better and receive associated general education and course credit. Students who receive below a B-, but still wish to declare a major are required to retake the course before admission into the bachelors program is approved.

Since inception of the three individual courses, 676 students have enrolled in PH 201, 265 students have enrolled in PH 202, and 237 students have enrolled in PH 203. Enrollment in PH 201 typically ranges from 75 to 100 students per semester, and enrollment in each PH 202 and PH 203 typically ranges from 30 to 40 students per semester. PH 201 is a large course, open to all students on campus, and is approved by the institution as meeting 3 credits of a 6 credit general education DS (Diversification in Social Sciences) requirement^4^, which all undergraduate students need prior to graduation. It attracts prospective undergraduate public health students, prospective graduate-level public health students, as well as students from other programs (such as biology, kinesiology, or psychology) looking to compliment their coursework, or any undergraduate in search of a DS requirement.

## Course design and pedagogical format

### Introduction to public health (PH 201)

The first course in the series is intended to give students a broad overview of public health, centered on concepts related to epidemiology, health promotion, and disease prevention. Textbook readings are assigned (25). Throughout the course, students are actively engaged in discussions and activities that promote a greater understanding of public health as a system, as well as its interdisciplinary connections to other health care fields. Critical thinking and analysis of important public health issues are also emphasized throughout the semester.

Lectures are interactive, and each class session strives to incorporate at least one activity or small group discussion with report-back, transitioning into a large group discussion. Students complete worksheets related to public health milestones (26) and public health careers (organized by the ten essential functions of public health) and then discuss them in small groups. Students conduct a mock disease outbreak investigation, working in small groups on a worksheet-assisted, scenario-based activity applying basic epidemiological investigation skills and calculations (e.g., relative risk) taught in a previous class session. On two separate occasions during the semester, students work in small groups to prepare, present, and defend an assigned position on an ethical issue in public health, one centered on environmental health and the other on health policy. This education technique, known as deliberative democracy, has been shown to increase students' ability to evaluate and synthesize information and to enhance civic engagement (27, 28). Past debate topics have included climate change, cap-and-trade of carbon emissions, tobacco control on campus, and e-cigarette regulation.

To assess mastery of the foundational domains and cross-cutting concepts, students take an open-book/open-note cumulative final exam. This approach is used to reduce student anxiety, and it has been found to increase student use of and appreciation of textbooks and notes, supporting the development of good research skills rather than memorization (29).

### Public health issues in hawai‘i (PH 202)

This course focuses primarily on application of general public health concepts and tools to public health issues relevant to the state. There is as yet no textbook on this topic, but relevant readings are assigned, and blogs are expected to reflect material from these readings, as well as course activities.

The course starts by providing the historical context for public health in the state by reviewing the history and impact of colonization and immigration in the state. In addition to public health achievements of the state, several controversial public health decisions are discussed, including the burning of Chinatown to eradicate the plague and the displacement of people with Hanson's disease. Guest speakers contribute by presenting on specific, community-based and culturally relevant programs that address the state's unique populations, problems, and policies.

PH 202 emphasizes community-based learning. For example, the students learn and practice using the phone/tablet application EpiCollect to gather “public health in action” photos and link them by GPS to a map. They also conduct a community windshield assessment to gather data on the built environment and identify elements that encourage or discourage health within students' neighborhoods. They also participate in one or more community work days of their choice, such as tending a community garden, cleaning up a stream or beach, or restoring a cultural site.

Classroom assignments challenge students to work collaboratively to present on public health stories in the local news, to create a culturally competent public service announcement, to design an ideal built environment, and to propose and defend ways to balance the state's budget. In lieu of a final examination for PH 202, students prepare and present a public health challenge and success in the state, including background, description, key stakeholders, public health significance, potential solutions, and what public health professionals outside of the state can learn from us.

### Introduction to global health (PH 203)

This course is designed to facilitate student application of public health concepts and skills to global public health issues. Guided by a textbook (30), students learn about public health challenges faced by different countries, for example poor air quality in China, the rising prevalence of non-communicable diseases in India, water and sanitation issues in Myanmar and Iraq, and problems facing indigenous populations around the world. Equally as important, they learn about the linkages between health, economic, and social development in a global context, the relationship between disparities in health and disparities in socio-economic indicators, and the impact of globalization on health. Population-based public health interventions to address this wide array of health challenges are discussed as well, including the role of community-based, interdisciplinary, and transdisciplinary approaches to address global health issues.

Assignments include a scenario-based, role-play activity in which students are assigned different roles (e.g., governmental leadership, international aid organizations, and private citizens) and asked to investigate and respond to a real-world scenario involving health policy, environmental health, and indigenous health. One year, for example, students were asked to find, consider, and debate information on the effects of climate change and sea level rise on the Pacific nation of Kiribati. Throughout this exercise, students are expected to apply critical thinking skills and work collaboratively to develop an action plan on how to proceed, while balancing the priorities of multiple perspectives.

The final project is an individually written paper on a global health topic or issue, in which the student describes the problem, discusses potential causes, reviews actual, and potential responses, outlines the public health implications of the problem, and makes recommendations for action.

### Coverage of CEPH requirements

The activities of three introductory courses are cross-walked with the CEPH requirements for the nine foundational domains outlined in criterion D10 (Table 1), the 12 cross-cutting concepts and experiences outlined in criterion D13 (Table 2), and the two foundational competencies outlined in criterion D11 (Table 3). As shown in Table 1, most aspects of all domains are introduced in PH 201 and covered in more depth in PH 202 and 203. Obvious exceptions are the science of human disease, which is covered in a required public health biology course, and evidence-based interventions and project implementation, which is covered in the capstone course series (13). The lectures and activities of the introductory series also cover the CEPH-required cross-cutting concepts and experiences (Table 2) and the two foundational competencies (Table 3). These competencies are reinforced in later courses and demonstrated by students through the capstone series.

**Table 2:** Bachelor's degree cross-cutting concepts and experiences (CEPH criterion D13).

	**Type^a^**	**1**	**2**	**3**	**4**	**5**	**6**	**7**	**8**	**9**	**10**	**11**	**12**
**PH 201 Introduction to PH**													
Policy process	L/D	x						x	x				
Behavior change and determinants	L/D		x										
Defining indigenous populations	L/D				x								
Measures of disease frequency	A										x		
Mock outbreak investigation	A			x									x
PH career with functions of PH	A						x			x			x
PH milestone	A/P	x					x					x	
Deliberative democracy-health policy	A/P	x		x		x		x			x	x	x
Deliberative democracy-env health	A/P			x		x		x			x	x	x
Weekly blogs	W						x						
**PH 202 Public Health in Hawai‘i**													
Raising tobacco possession age to 21	L/D	x							x				
Suicide prevention among local youth	L/D			x									
Tourism and influenza	L/D			x							x		
Impacts of sea level rise	L/D											x	
Community workday participation	A				x		x						
Windshield assessment of built env	A					x					x		
PH in Action photo collection	A/P				x		x				x		
Design an ideal built env	A/P				x							x	x
Culturally appropriate PSA	A/P			x	x								x
PH in the news	A/P		x					x					x
PH successes and challenges	A/P			x	x	x				x		x	x
Balance the state budget	A/P	x		x		x						x	x
Weekly blogs	W						x						
**PH 203 Introduction to Global Health**													
Water and sanitation in Myanmar and Iraq	L/D	x							x				
Non-communicable diseases in India	L/D		x										
Global burden of HIV/AIDS	L/D										x		
Air quality in China	L/D											x	
Indigenous culture and health impacts	L/D				x								
Role-playing scenario	A			x									x
Global health topic presentation	P/W	x		x			x			x		x	
Weekly blogs	W						x						

a Type, L/D, lecture/discussion; A, activity; P, presentation; W, written work.

*1, advocacy for protection and promotion of the public's health at all levels of society; 2, community dynamics; 3, critical thinking and creativity; 4, cultural contexts in which public health professionals work; 5, ethical decision making as related to self and society; 6, independent work and a personal work ethic; 7, networking; 8, organizational dynamics; 9, professionalism; 10, research methods; 11, systems thinking; 12, teamwork and leadership*.

**Table 3:** Bachelor's degree foundational competencies (CEPH criterion D11).

		**Communication: ability to communicate PH information:**				**Information literacy ability to:**			
	**Type^a^**	**C1 in written form**	**C2 in oral form**	**C3 with diverse audiences**	**C4 through a variety of media**	**IL1 locate PH information**	**IL2 use PH information**	**IL3 evaluate PH information**	**IL4 synthesize PH information**
**PH 201 Introduction to PH**									
Weekly blogs	W	x							x
Health communication	L/D			x	x				
Information resources	L/D					x			
Critical thinking	L/D							x	
Mock outbreak investigation	A				x		x	x	x
PH careers within functions of PH	A		x				x		
PH milestone presentation	A/P		x				x		
Deliberative democracy-health policy	A/P		x			x	x	x	x
Deliberative democracy-env health	A/P		x			x	x	x	x
**PH 202 Public Health in Hawai‘i**									
Weekly blogs	W	x						x	
Raising tobacco possession age to 21	L/D			x	x		x		x
Suicide prevention among local youth	L/D		x	x			x		
Tourism and influenza lecture/discussion	L/D			x			x		
Impacts of sea level rise lecture/discussion	L/D				x		x		
PH in action photo collection	A/P				x	x	x		
Community workday participation	A			x	x				
Windshield tour of built env	A					x			
Design an ideal built	A/P				x		x	x	x
Culturally appropriate PSA	A/P			x	x		x		x
PH in the news	A/P		x			x	x	x	x
PH successes and challenges	A/P			x	x	x	x	x	x
Balance the state budget	A/P	x		x			x		x
**PH 203 Introduction to Global Health**									
Weekly blogs	W	x							
Water and sanitation in Myanmar and Iraq	L/D	x		x			x		x
Non-communicable diseases in India	L/D			x			x		
Global burden of HIV/AIDS	L/D			x			x		
Air quality in China	L/D			x			x		
Indigenous culture and health impacts	L/D			x			x		
Role-playing scenario	A			x		x	x		x
Global health topic presentation	P/W	x				x	x	x	x

a *Type, L/D, lecture/discussion; A, activity; P, presentation; W, written work*.

## Assessment procedures

Average scores of the final exam in PH 201 over the past two academic years, (four semesters, *n* = 374 students), ranged from 81.66 to 88.41%. These data, combined with data from rubric-graded class assignments throughout the three introductory core courses, suggest that students are mastering the course content and skills. Data from mid- and end-of-semester course evaluations, as well as data from graduation exit questionnaires and student focus groups, additionally suggest that students appreciate being challenged to apply concepts from introductory public health to local and global settings in the later courses. Feedback from faculty of advanced undergraduate public health coursework suggests that the introductory series prepares majors for success in more-advanced public health coursework and service learning.

## Discussion

The undergraduate public health introductory core course series appears to successfully provide a broad-based foundation of public health knowledge for students. Furthermore, the three-course structure allows students to be exposed to core concepts in an overview course and then to have these concepts reinforced in the subsequent classes through small-group inquiry-based activities that guide students through the application of these key concepts to both local and global settings, a pedagogical technique that has been shown to help students master complex material (9, 23). The series addresses CEPH requirements for undergraduate education, and evaluation data suggest that students are gaining required knowledge and skills and that they enjoy the courses, as evidenced by individual course evaluations as well as exit surveys and focus groups conducted at the time of program completion.

As with all successes come challenges. These courses, and the BA PH program, have grown in popularity since 2014, and larger class sizes have required adjustments. PH 201, which serves as a gateway to the major, now accommodates 100 students per semester, while the class sizes of PH 202 and 203 have expanded to 40–50. As a result, small-group activities are conducted in groups of four or five, instead of in pairs. However, larger class size increases the diversity of students, thought, and creativity. Today, students in PH 202 formulate six to eight culturally appropriate public service announcements, whereas smaller classes created only three or four.

Articulating with local community colleges provides another opportunity to address program expansion (31, 32). Because these courses are at the 200 level, they can be offered by community colleges as part of a 2-year degree. One challenge experienced, however, is that community colleges may not have the resources to offer the full sequence of three courses. Most community colleges in the state do have the capacity to offer at least the first course, but such discussions with community college faculty are still in early stages. The proposal is for community college instructors to be provided with instructional materials, as well as grading rubrics, and trained by the primary instructor of the course at the UH Manoa campus prior to offering a course at the community college-level. Community college graduates may then choose to transfer to the UHM campus to complete the BA PH or take advantage of distance education options. The deliberate integration of material from PH 201 into the two following courses allows for continuity and expansion as students transition from their community college to the 4-year BA PH program at the UHM. The active learning components of the activities in these courses can also help students integrate into a new program and a new major, and should help strengthen their comprehension of the more complex material covered at this level (33).

The UHM experience demonstrates that creating thoughtful curricular linkages between students' introductory course in public health concepts and immediate follow-on courses in public health issues, (such as global health and regional health concerns), enhances the depth of student learning in the critical first year of public health studies. The deliberate articulation of content between these courses provides critical opportunities for students to engage with core public health concepts well beyond what can be achieved through individual, stand-alone courses. Since most public health programs already include introductory, global health, and regional health courses within their curricula, developing this foundational series is relatively easy and inexpensive, requiring simply the thoughtful re-tooling of existing courses and course progression plans.

For programs unable to accommodate such a model into their schedule, using elements of the framework described in this paper may serve as an opportunity to incorporate local or global health examples or course activities into existing coursework, and may help further engage students in public health and active learning pedagogy.

This foundational public health series provides a much-needed model of an introductory course series that is easy to implement, meets CEPH requirements, is well-received by students, and prepares students well for upper-division public health courses.

## Author contributions

DN-H conceived the initial concept, drafted manuscript, and provided critical review and revision of individual sections. MT assisted in program design and development, assisted in development of initial manuscript draft, and provided critical review and revision. LK assisted in program design and development, aided in development of manuscript, and provided critical review and revision. OB aided in development of manuscript, and provided critical review and revision of individual section. KB provided administrative perspective, aided in development of manuscript, and provided critical review and revision of individual sections.

### Conflict of interest statement

The authors declare that the research was conducted in the absence of any commercial or financial relationships that could be construed as a potential conflict of interest.

## Footnotes

^1^https://www.aspph.org/connect/data-center/.

^2^http://www.aspph.org/educate/framing-the-future/.

^3^https://manoa.hawaii.edu/about/.

^4^http://www.catalog.hawaii.edu/corerequirements/index.html.
